# Subtitling Arabic profanities into English and that aggro: the case of *West Beirut*

**DOI:** 10.1016/j.heliyon.2022.e11953

**Published:** 2022-11-28

**Authors:** Mohammad Ahmad Thawabteh, Amer Al-Adwan, Amna Shqair

**Affiliations:** a Al-Quds University, Jerusalem, Palestine; b Hamad bin Khalifa University, Qatar

**Keywords:** Audiovisual translation, Subtitling, Taboo language, Subtitling strategies, DTS

## Abstract

The present article explores one of the most unexpected and unpredictable changes of taboo language by language users and its translation into English as can be illustrated in the analysis of a Lebanese movie entitled *West Beirut.* The article first argues that taboo language is surely far more difficult than any other types of language to deal with in subtitling. Such a language is inelegant, but so confusing insofar as subtitlers are concerned, apparently due to the degree of social acceptability of the profanities by various cultures on the one hand, and to the technical restrictions related to the subtitling process on the other. The article shows that in an attempt to ensure mastery of the intricacies resulted from the use of obscene-loaded language and technical constraints associated to them, subtitlers use a considerable number of translation strategies. The article adopts a two-integrated approach: Toury's (1995) product-oriented Descriptive Translation Studies (DTS) and ‘coupled pairsʼ to analyze the original dialogue and its English equivalent. The findings reveal that the subtitlers have resorted to six translation strategies while dealing with excessive taboo language density, namely cultural substitution (adaptation), literal translation, euphemism, omission, reformulation and change in the semantic field. It might then be concluded that utilizing some of these strategies have resulted in distortions of the original dialogue, while others have managed to reach its intended audience. Finally, the article highlights that cultural considerations play a major role in determining the translation strategies and their frequency in communicating taboo language into other cultures.

## 1 Introduction

The inception of technology was characterised by inchoativity. The digital and technological revolution in the 1990s has dramatically brought people closer and changed the way they seek information and education on several levels (Gamal 2007, p. 78). Obviously, the bleeding-edge of technology insofar as Translation Studies is concerned has been the advent of Audiovisual Translation (AVT) in the past few decades. “AVT is closely linked to technology and any new advances are bound to have a knock-on effect on the discipline, particularly on subtitling. The Internet has been one of such advances” (Díaz Cintas and Anderman 2009, p. 15). Most of the people nowadays rely on screens to obtain their pertinent information, to do businesses and to seek entertainment, among many others (Gamal 2007, p. 78). This advancement in technology has dramatically changed the nature of social interaction, encouraging people to explore other societies and to learn more about their cultures. This, in turn, has created a remarkable and constant demand on different modes of translation, especially AVT.

Matkivska (2014, p. 38) defines AVT as the transfer of the verbal components of audiovisual work and products from one language into another. In his definition, Khuddro (2018, p. 21; italics added) points out a kind of solidarity with Matkivska's definition that “AVT is concerned with the transfer of *multimodal* and *multimedial* texts into another language and/or culture.” Khuddro (2018, p.10) further adds that AVT “includes both audio translation, either dubbing or voice-over mode, and visual translation for captions/supers or subtitles using television, cinema, and other devices such as computers and mobile phones that are spreading fast in this digital age.” Therefore, AVT modes, particularly subtitling, are considered to be a dramatic development in the field of Translation Studies (Munday 2009, p. 275). Nevertheless, a plethora of studies on this domain have demonstrated that translation practitioners encounter a composite of multifarious difficulties and restrictions whilst translating audiovisual materials, some of which are closely related to technical constraints (spatial and temporal), while others are related to linguistic and cultural differences (see for example, Baker 1998; Al-Adwan 2015; Furgani 2016; Gamal 2007). In this regard, Neves (2004, p. 135) aptly remarks:

In [AVT] the problems which arise are somewhat similar to those of literary translation with the extra stress that the fidelity factor is dictated by constraints that lie beyond words or languages. Whereas in written translation fidelity lies in two extreme points, the source-text or the target-text, in [AVT] fidelity is particularly due to an audience that […] is in need of communicative effectiveness, rather than in search of artistic effect-as is the case in literary translation- or of exact equivalence- as happens with technical translation.

Other constraints are in principle closely related with culture— “the way of life and its manifestations that are special to a community that uses a particular language as its means of expression” (Newmark 1988, p. 94; see also Tomaszkiewicz 2010, pp. 93–106). In this, language and culture are deemed inseparably intertwined. Each language and culture have their one-of-a-kind systems of words and expressions in the treatment of taboo language (Ávila-Cabrera, 2016, p. 28).

The current article carefully examines the asymmetrical system of linguistics between Arabic and English, namely the expression of offensive and taboo language. Ávila-Cabrera (2016, p. 28) believes that “[o]ffensive and taboo language exists in the majority of cultures although the acceptability of this type of linguistic register differs according to the type of society, culture, beliefs and the like”. Broadly speaking, offensive language, in the words of Ávila-Cabrera (2016, p. 28), refers to the linguistic terms and expressions that are often deemed insulting and/or derogatory. Dealing with such a language, the fundamental task of the translator at work seems to unequivocally be easier in printed translation than AVT in view of a number of translation strategies available to him/her, such as definitional extensions, paraphrases, translation footnotes, etc. In AVT, however, employing such strategies seems to be bounded by technical, linguistic and cultural restrictions (Łabendowicz 2012, p. 2). Therefore, in an attempt to overcome these constraints, translators may adopt other strategies such as literal translation (direct transfer), substitution, adaptation, euphemisms and many others (Łabendowicz, 2012, p. 2).

## 2 Audiovisual translation (AVT)

“A quick perusal of traditional television programmes or cinema guides will testify to the growth and importance of the media and the need for audiovisual translation (AVT) in most countries” (Díaz Cintas and Anderman 2009, p. 2). The world nowadays is mildly influenced by the information broadcast and circulated by the media. Needless to say, new forms of intercultural communication have emerged, giving rise to using new methods and modes of translation (Matkivska 2014, p. 38). AVT is an umbrella term that encompasses new sub-modes of translation (i.e., voice-over, dubbing, subtitling, etc.) all of which are deemed to be a basic progression in the field of Translation Studies (Munday 2009, p. 275). AVT is considered as an interdisciplinary field that lends itself to several overlapping areas of knowledge such as media translation, multimedia translation, multimodal translation and screen translation, all of which set out to cover the interlingual rendering of verbal language when transmitted and accessed both visually and acoustically through some kind of electronic device most of the time (Munday 2009, p. 141).

In dealing with audiovisual products or materials, translators do not only work solely with texts, but they also should attempt to consider other semiotic features of communicative potential of subtitles. Similarly and as a necessary consequence, they deal with dialogues, comments, sound effects, images and atmosphere of the video (Matkivska 2014, p.38). According to Baker (1998: 245), there are four major channels of information for audiovisual materials that should be taken into account without compromising source dialogue subtlety: (1) the verbal auditory channel, e.g., dialogue, background voices, and sometimes lyrics; (2) the non-verbal auditory channel, e.g., music, natural sound and sound effects; (3) the verbal visual channel, e.g., superimposed titles and written signs on the screen; and (4) the non-verbal visual channel, e.g., picture composition and flow.

### 2.1 Subtitling

It is axiomatic that subtitling is one of the most popular modes of AVT, roughly defined as transferring filmic dialogue in one language into written subtitles displayed in cinema or on television screen, with synchronization of acoustic elements and visual elements into full consideration. It usually refers, in the words of Díaz Cintas (2001, p. 23), to a linguistic practice that aims to offer a written text at the bottom of the screen. More precisely, subtitling is described as the “transcriptions of film or TV dialogue, presented simultaneously on the screen [and] usually consist of one or two lines of an average maximum length of 35 characters” (Baker 1998: 245; see also De Linde and Kay 1999, p. 6).

Depending on the mode of projection, subtitles can be either superimposed on the film itself (open subtitles) or displayed based on the selection of the viewer to project them on the screen (closed subtitles) (Munday, 2009, p. 148). On the difference between the skills required from a translator and those required from a subtitler, Kruger (2008, p. 8) notes that “subtitling requires all the skills that other modes require in terms of text analysis, subject expertise, and language awareness of context, quality control and so forth.” Yet, the subtitler has to be capable of applying all these skills within any rigid constraints of time and space while abiding by certain conventions of quantity and form (Furgani 2016, p. 9).

It ensues, therefore, that the subtitler's job is significantly more difficult than the translator's primarily due to technical competence; “use of software, line breaks, positioning on the screen, time and space restrictions, use of italics, etc.” (Skuggevik 2009, p. 198). For time and space restrictions, for example, the content of the dialogue has to be condensed and displayed on screen in synchrony with the original dialogue (Furgani 2016, p. 13).

### 2.2 AVT in the Arab World

Traditionally, subtitling has been primarily the most preferred form of AVT in the Arab World, especially in Egypt, where cinema and film production emerged as a new reality at the beginning of the 20th century (Gamal 2007, p. 79). However, thanks to the unambiguously fear pertinent to the high quality of the production of American films compared with the immature industry in the Arab World, Egyptians decided against dubbing (Gamal 2007: 79). Having launched state-run television in most Arab countries, e.g., Iraq in 1956, Lebanon in 1959 and then the rest of other Arab countries, subtitling of foreign programmes began to nourish and viewers started appreciating this mode of translation (Gamal 2007, pp. 79–80).

By the 1990s, there was a rapid expansion in the number of satellite channels. To fill the broadcasting hours of these channels, they resorted to foreign programmes, creating a persistent need for employing different forms of AVT, be subtitling, dubbing or voice-over (Al-Adwan 2019, p. 64). Today, there are hundreds of satellite channels in the Arab World, some of which are foreign ones such as Discovery Channels, National Geographic and Disney Channels. Consequently, there is a high demand for AVT modes, especially subtitling (Gamal 2007, p. 80).

## 3 Offensive language and taboo words

Perhaps it is useful to provide insights into dysphemism. It is true without a shadow of a doubt that dysphemism belongs to an offensive language and taboo words. By definition, dysphemism according to Allan and Burridge (1991, p. 26), is “an expression with connotations that are offensive either about the denotatum or the audience or both, and it is substituted for a neutral or euphemistic expression for just that reason”. The use of offensive language and taboo words (be written or spoken) is not a new phenomenon to fill in the likely communicative loss in everyday use of the language. In fact, there is widespread evidence that it is ubiquitous in all cultures, and is usually employed for a special, often forbidden and/or prohibited terms with magic and myths (Ávila-Cabrera, 2016, p. 3). Known as obscene, profane language, offensive language, or imprecatives, such language and taboo words are used to refer to the linguistic terms and expressions that are often considered pernicious, insulting and derogatory. Likewise, taboos are related to the terms that are deemed inappropriate and doubtlessly unacceptable in formal contexts, cultures, languages and/or mediums where they are made (Ávila-Cabrera, 2016, p. 28). They can be classified into various categories: sexual and scatological obscenities, ethnic and racial slurs, religion, physical and mental disabilities, blasphemy, slang and vulgarities of all kinds (Pardo 2013, p. 123). Such expressions and other similar ones are usually used to express the speaker's strong emotional feelings such as showing anger, contempt, or vexation (Hawel 2019, pp. 425–426).

It must also look at offensive language and taboo words as part and parcel of the very nature of culture despite the fact that the social acceptability of these words varies depending on the type of society, cultures and beliefs, religion, history and social structure (Ávila-Cabrera, 2016, p. 29). Battistella (2005, p. 83) states that “what seems clear overall is that the notion of offensive language is a variable one, shifting over time and affected by social, historical, political, and commercial forces”. Thus, plausible judgement whether or not an action or an expression is deemed taboo or offensive is basically difficult to pin down because it is not the same for each society, but is conventionally considered society-, behaviour- and culture-specific (Pardo 2013, p. 123). Insofar as Arabic culture is concerned, for instance, it will be helpful to note that it is generally known to be conservative due to the impact of religion on society. Consequently, it might be unexpected and unusual to use taboo words in the Arabic media. This leaves translators with only very few options when they encounter these expressions in audiovisual material (Al-Yasin & Rababʻah 2019, p. 3).

It ensues, therefore, that it is probably true that conservatism is a quintessential part of Arab cultures, but a rare exception to this state of affair might be observed in some Arab cultures, e.g., the Lebanese culture, which might be frowned upon as progressive.

## 4 Subtitling strategies

Due to the invariable difficulties of translating a stretch of an utterance between remote languages and cultures, the translator should be optimally fastidious to properly overcome these difficulties. Hence, devising an appropriate strategy turns out to be not only feasible and necessary, but is also a dire need. Scott-Tennent et al. (2000: 108) define translation strategies quite non-restrictively as “the steps, selected from a consciously known range of potential procedures, taken to solve a translation problem which has been consciously detected and resulting in a consciously applied solution”. This is as true of translation as it is of subtitling. In dealing with audiovisual material, the subtitler may face several restrictions, some of which are inherently of technical nature whilst others are closely associated with linguistic realities and, most importantly, culture-bound expressions as is in the case of offensive language and taboos. This continues to impose a heavy burden on the translator's shoulders, and thus a number of appropriate strategies have to be adopted in order to surmount any potential difficulty. The most appropriate translation strategies for this kind of language, Baker (1992) argues, are softening and literal translation. By the same token, Khalaf and Rashid (2016, p. 295) succinctly suggest six basic sets of strategies for the translation of taboo terms insofar as Arabic is concerned, i.e., deletion, register shift and the use of archaic words, change in the semantic field, the use euphemistic expressions, generalization and linguistic substitution.

Subtitle-wise, Díaz Cintas and Remael (2014) believe that there are other strategies that cannot be ignored when subtitling offensive language and taboos: (1). literal translation, a strategy that involves transferring the word or the phrase from the SL into the TL while keeping the idiom and the grammar of the SL; (2) calque translation, a verbatim translation of a word or expression in a way that is unusual or does not sound idiomatic in the TL; (3) reformulation, used for paraphrasing linguistic structures and expressing them in a different way in the TL; (4) compensation, a strategy that involves overtranslating a term or adding equivalent information in the TL. Yet this method is hard to apply in the subtitling environment owing to the time and space restrictions; (5) omission, a common strategy in subtitling due to technical limitations. Therefore, it is sometimes preferred to omit certain elements (cited in Ávila-Cabrera, 2016, p. 41), and (6) cultural substitution, also referred to as adaptation by other scholars. This strategy, according to Fawcett (1997, p. 39), occurs when an expression or a phrase specific to one language or culture is expressed in a totally different way, that is more suitable and familiar to the target culture. Therefore, it is a good choice when translating taboo words as they are considered to be culture-specific trait of Arab culture.

In addition to these strategies, Jing-Schmidt (2019, p. 3) suggests another strategy, namely using euphemism to translate offensive language and taboo words, roughly defined as “the use of deliberately indirect conventionally imprecise or socially ʻcomfortableʼ ways of referring to taboos, embarrassing, or unpleasant topics.” Arguably, this strategy could be one of the most suitable ones for translating such expressions as it helps to lessen the degree of profanity of these offensive expressions.

## 5 Methodology

Product-oriented Descriptive Translation Studies (DTS) and Toury's (1995) “coupled pairs” approaches constitute the methodological basis for the data analysis of the current study. Whilst the former encourages translators to “delve into translation as cultural and historical phenomena, to explore its context and its conditioning factors, to search for grounds that can explain why there is what there is” (Hermans 1999: 5) and focuses on the description of the product, i.e., the English subtitles of the film, rather than the process or the function, the latter entails a textual analysis and a comparison of the ST and TT in order to identify the relation between corresponding segments in the two texts (see Munday 2009, p. 175). Using these tools, the data analysis shows that the subtitler/translator opted for the following strategies when dealing with offensive language and taboo expressions: substitution, literal translation, change in the semantic field, euphemism, reformulation and omission.

The overall objectives of the study are to identify the obscenities in the Lebanese Movie *West Beirut*, and to push further investigation ahead to ascertain whether or not the subtitling strategies employed by the subtitler in translating the movie from Arabic into English are effective. It also attempts to find out whether or not the subtitler has succeeded in conveying the original sense of the offensive language and taboo words in the movie in light of various kinds of technical, linguistic and cultural restrictions.

### 5.1 Data of the study

This case study investigated in the current paper is a Lebanese film entitled *West Beirut* and its English subtitles with a particular focus on offensive and taboo references. It also sheds light on the strategies that subtitlers have employed in subtitling such language, usually replete with cultural and technical constraints.

*West Beirut* is a 1998 Lebanese film written and directed by civil war-meister Ziad Doueiri with an eye on daily details of the war that sent Lebanon plunging into anarchy, violence and bloodshed. The blockbuster is a highly autobiographical account for the grinding civil war that erupted in 1975. As a cliff-hanger, the film is an exposé of the war, teaming with obvious stories about the war in Beirut which was partitioned into the Muslim West Beirut and the Christian East Beirut. The film shows the chronic economic, social and political malaise due to that partition and how people were suffering from changes in an unfamiliar milieu. The film was characterized by the use of offensive language and taboo words by the film's characters throughout the events. An English subtitled version of the film is available on Netflix, an American media-services provider and production company in California, the USA.

### 5.2 Significance of the study

This study may be considered important as it copes with a very problematic area of culture that has a bearing on offensive language and taboo words. Unlike previous studies (e.g., Bhais 2011; Al-Adwan 2015; Khalaf and Rashid 2016; Hawel 2019; Al-Yasin; Rababʻah 2019) that dealt with the subtitling of English swear and taboo references into Arabic, the current paper deals with how subtitlers treated these problematic utterances from Arabic into English in light of technical constraints usually associated with subtitling.

It goes without saying that few studies (e.g. Al-Adwan and Thawabteh 2021) investigate the subtitling of Arabic offensive language into English, as this linguistic phenomenon is more common in the latter. Thus, this paper aims to pave the way for more studies in this marginalized line of research in Arabic AVT, namely Arabic-English subtitling.

## 6 Discussion and data analysis

In this section, we shall go through illustrative examples of the strategies adopted in the film with a fine-tooth comb in order to diversify and corroborate our argument. The analysis of the English subtitles shows that offensive and taboo utterances have fallen within the scope of a set of references, mainly related to animals, prostitution, sexual organs and relations, faeces, slang and vulgarities. For the sake of the study, a taxonomy of the strategies apparently utilized in the film is made, comprising of a set of strategies: cultural substitution (i.e. adaptation), literal translation, euphemism, omission and change in the semantic field (see Díaz Cintas and Remael, 2014).

### 6.1 Cultural substitution (adaptation)

In carefully scrutinising Text 1, the commonly adopted strategy (i.e., cultural substitution) has largely been used to deal with cultural expressions germane to a particular culture and absent from the other. Such expressions are akin to prostitution, threatening, sex and insulting others using offensive references that have no equivalence in the TL and culture, as can be clearly seen in the following example:

**Table:** Text 1

ST	ولا أبو خضر
	فزرة اللي تفزرك
TT	Hey, Abu Khodor
	Goddamnit!

In this example, the speaker shouted an insult at the addressee aggressively ‘فزرة اللي تفزرك’ (lit. ‘May your body protrude seamlessly’) usually used in response of insouciance on the part of the addressee. In terms of strategy, conspicuous meticulous attention to such culture-specific item is paid whereby the translator has employed cultural substitution, i.e., ‘goddammit!’ to inflict on the target language audience humiliation. Versteegh (1997, p. 423) points out that the use of swear word denotes “a direct approach to the cursed person”. In the words of Khalaf and Rashid (2016), it is a kind of ‘linguistic substitution’ that is also conducive to cultural substitution as well (see also Díaz Cintas and Remael, 2014). It is worth noting that the very rude informal translation, however, is explicit with obscene connotation.

Similarly, Text 2 shows cultural substitution as a strategy for peculiar attention-grabbing insult in Arabic.

**Table:** Text 2

ST	ابن الكلب سبيرو عم بدفعنا 10 ليرات هالايام ليظهر الفيلم
TT	The son of a bitch Spiro charges ten bucks a roll

A fairly obvious claim to make is that the speaker in the utterance above has shouted in a very loud voiceابن الكلب (lit. ‘son of a male dog’) to express a twinge of furious feeling in view of ‘charges ten bucks a roll’. In this example, cultural substitution is apparently used to deal with such revulsion by employing ‘a bitch’ (female dog) in English, a strategy that would seek to attain natural attitudinal values.

### 6.2 Literal translation

As can be observed from the analysis of the data, literal translation might be said to constitute another frequently occurring strategy. To build up a more accurate and detailed picture of how this strategy is employed, consider the following example:

**Table:** Text 3

ST	الله يخرب دياركم متل ما خربتوا ديارنا
TT	May Allah destroy your homes like you did to ours.

In this example, the speaker bawled الله يخرب دياركم متل ما خربتوا ديارنا (lit. ‘May Allah destroy your homes like you did to ours.‘) with such a drawn-out voice to express the state of scumbag. The Arabic utterance occurs at turn boundaries of the text usually with a view to showing an appeal to Allah, to distinctively express the meaning of ʻangerʼ and annoyance that can functionally mean ‘there'll be hell to pay’. The subtitler has resorted to literal translation strategy as there is no formal equivalence in English for such an expression, thus unlikely to be propitious in a general sense. The sense of the SL has been, however, maintained and the intended message has been conveyed.

**Table:** Text 4

ST	.وليه إنت أهلك ما ربوك
TT	Your parents didn't raise you.

In text 4 above, straightforward literal translation is likely to be appropriate as the denotative meaning is captured nicely as ‘raise’ denotes looking after a child. Nevertheless, in no way can it be claimed to do the trick— the translation of idiolectأهلك (lit. ‘your parents’) into mere ‘parents’ falls short pragmatically. To restore meaningfulness, perhaps ‘mum' and ‘dad’ or simply ‘folks’ can be an option.

### 6.3 Euphemism

Based on the data analysis, euphemism is another commonly used strategy for subtitling taboo words. As pointed out earlier, euphemism, according to Allan and Burridge (1991), entails substituting dispreferred or dysphemistic expressions with other softened ones as a way to avoid losing face (cited in Thawabteh (2012), p. 147; see also Khalaf and Rashid (2016), p. 295 and Jing-Schmidt (2019), p. 400). As a result, the subtitler has resorted to this strategy to attenuate the bad effect that these words may have on the viewers as can been seen in the following example:

**Table:** Text 5

ST	!تصور بدو يفيقنا من ط∗∗ الصبح لنصلي
TT	He wants us up at the crack of dawn to pray

Gritting his teeth, the speaker adeptly rubbed off on and berated the addressee. A close look at this example shows that the Arabic circumlocutionary euphemism is used by the speaker with a view to groaning about rousing early in the morning. In Arabic, “the native speakers’ recourse is to use some euphemistic formulas to mitigate that horrible meaning” (Al-Qadi 2009: 18) and this might be done as Ath-Tha’alibi, 1972 claims, by means of equivocation to express obscene situations.

More to the point, language variety plays an active role in toning down the original utterance. Modern Standard Arabic, for example, has a propensity to be more euphemism-like whereas slang tends to be dysphemism-like (see also Thawabteh 2017, p. 567). The Lebanese dialect might then have a tendency towards obscenity as the data of the study succinctly shows.

In Text 5 above, the speaker shrieks obscenities at the addressee, but the translation was softened using gentler words— first letter with two asterisks, i.e., ط∗∗(lit. ‘asshole’, a letter that is very embarrassing and offensive since it indicates a sexual organ, i.e., a person's anus. The English translation has no offensive effect on the viewers as “at the crack of dawn” is used to emphasize doing something very early in the morning.

A telling example as to the use of euphemism can be observed in Text 6 below whereby an extreme obscene Arabic itemبجم (lit. 'scum') is ameliorated into a less provocative attitude.

**Table:** Text 6

ST	شو بعملك بناس بجم بها البلد
TT	I can't stand it either!

### 6.4 Omission and change in the semantic field

Due to lack of equivalence and the high degree of obscenity, this strategy might be used. Al-Adwan (2015, p. 17) speaks of what is termed as ʻsemantic misrepresentationʼ:

This process relies crucially on shifts across various semantic fields in a particular context, leading to the production of a type of euphemism that is very specific to the context of translation. This device sacrifices the semantic content of the offending item in favour of a substitute, often derived from a different semantic field, which completely avoids the offensive reference.

For the sake of elaboration, consider Text 7 below:

**Table:** Text 7

ST	هاد عازوري بعد لبلّعه صرمايتي
TT	TT: I'm going to kick his ass

In pursuit of effective discoursal thrust, the SL speaker has a lackadaisical attitude towards the addressee, and talks disparagingly about the overall unpleasant situation. It is noteworthy that the vernacular Lebanese Arabic صرمايتي (lit. ‘my shoes’) has been omitted in the English subtitle. This might be because of the high degree of profanity in this word or lack of equivalence in the TL. Furthermore, a substitute, i.e., ‘ass’ derived from a different semantic field has been used.

**Table:** Text 8

ST	وليه إنت واحد شخاخ. وليه إنت واحد أزعر
TT	You're an impolite pigheaded pissball!

A cursory reading of text 8 shows a clear idiolectal feature underlying a sacrilege to offend the intended addressee hurling epithet i.e., شخاخ (lit. ‘exuding urination’). The semantic import in the TL accounts for denotative and connotative layers of the source text. To relay the necessary pragmatic thrust, the idiomatic ‘he is pissing himself’ might be an option.

### 6.5 Reformulation

Following an interpretive approach associated with a group of scholars known as the Paris School, Almanna (2013, p. 49) speaks of reformulation stage translation or interpreting “in which the interpreter/translator starts searching for an idiomatic means of expression that can render the sense of the original by complying with the usage and customs of the TL”. Consider again Text 7 above which is used to illustrate a totally different strategy, i.e., reformulation. That is to say, ‘kick his ass’ is utilized to meet the linguistic and cultural expectations of the TL audience. To further elaborate on this strategy, consider the following examples:

**Table:** Text 9

ST	!ولا وجع اللي يهريك هري
TT	May Allah spread pain all over you

In this example, the Arabic utterance that literally reads ‘You're writhing in excruciating pain!’ is so offensive. The subtitler opted for a kind of reformulation strategy to translate the Arabic sentence into English, mostly because there is no equivalence for it in English.

For more illustration, consider Text 10 below:

**Table:** Text 10

ST	!حد يطلعله لجوزي ينزل قطيعة يلي تقطعة وتوخذه
TT	Somebody go get my husband. The idiot

Giving a chortle, the speaker snubbed her husband in public describing him like an irresponsible imbecile. Such speaker's rudeness encroaches on her husband's negative face. It is worth noting that the subtitler has adopted a subtitling strategy that transcends reformulation whereby the sense of the original is retained in accordance with the TL socio-textual conventions. Orchestrating underlying socio-textual practice such a way in Arabic is particularly noteworthy as it has a pragmatic meaning potential of ‘idiot’.

## 7 Conclusion

Thus far, the discussion makes a plea for greater consideration to the subtitling of Arabic profanities into English and all difficulties and problems involved. The study revealed that both languages cut linguistic and cultural realities quite differently. Indeed, the obscenities are not understood the same way. In Text 8 above, whereas أزعر (lit. ‘troublemaker’) is much used in Arab culture, English employs an entirely complex cultural component (i.e., ‘pigheaded’), very much alien to Arab culture.

A workable taxonomy of the problems and the ensuing strategies in pursuit of optimal translation for subtitling profanities from Arabic into English were carefully investigated. Taking our cues from Toury's DTS and “coupled pairs”, we described the actual subtitling of taboo expressions impinging on a textual analysis and a comparison of the ST and TT. This article examined English profanity-loaded sequences drawn from the Lebanese movie *West Beirut*. It mainly aimed at revealing the strategies the subtitlers have opted for in transferring taboo references into English.

In terms of analysis, it can be concluded that translating such expressions is particularly challenging for subtitlers as they are culturally-specific and often do not have direct equivalents in English. To overcome this difficulty, the subtitler adopted a variety of translation strategies, namely cultural substitution, literal translation, euphemism, omission, reformulation and change in the semantic field. Some of these strategies, such as cultural substitution and literal translation helped the subtitler to maintain the sense and the effect of the ST in spite of the technical and cultural restrictions imposed on her/him. However, the rest of strategies such as omission and change in the semantic field have contributed to the loss of the ST sense and its intended impact. Overall, it could be argued that the subtitler has partly succeeded in maintaining the sense and effect of the SL through using these strategies. Arguably, English culture is more tolerable insofar as obscene language is concerned than Arabic culture. However, the English subtitles have often failed to present an accurate representation of the original characters and the nature of their interaction in the film.

A final conclusion is that the task of the subtitler is found to be fraught with peculiar perils, not only because of culture-specificity of taboo and obscene language, but also because of the strict limitation in employing subtitling strategies imposed by subtitling conventions, a point of interest for further studies.

## Declarations

### Author contribution statement

Mohammad Thawabteh and Amna Shqair: Conceived and designed the experiments; Performed the experiments; Analyzed and interpreted the data; Contributed reagents, materials, analysis tools or data; Wrote the paper.

Amer Al-Adwan: Performed the experiments; Analyzed and interpreted the data; Contributed reagents, materials, analysis tools or data; Wrote the paper.

### Funding statement

The Open access funding was supported by the Qatar National Library.

### Data availability statement

Data will be made available on request.

### Declaration of interests statement

The authors declare no conflict of interest.

### Additional information

No additional information is available for this paper.

## References

[bib1] Al-Adwan Amer (2015). Towards a model of euphemisation in Arabic subtitling. Arab World Engl. J..

[bib2] Al-Adwan Amer, Faiq Said (2019). Arabic Translation across Discourses.

[bib3] Al-Adwan Amer, Thawabteh Mohammad (2021). *Translation as a Set of Frames*, Almanna, Ali and Gu Chonglong.

[bib4] Al-Qadi Nasser (2009).

[bib5] Al-Yasin Noor., Rababʻah Ghaleb. (2019). Arabic audiovisual translation of taboo words in American hip hop movies: a contrastive study. Babel.

[bib7] Allan Keith, Burridge Kate (1991).

[bib8] Almanna Ali (2013).

[bib6] Ath-Tha’alibi Abu Mansour (1972).

[bib9] Ávila-Cabrera, Javier José (2016). Subtitling tarantino’s offensive and taboo dialogue exchanges into European Spanish: the case of pulp fiction. Rev. Lingüística Lenguas Apl..

[bib10] Baker Mona (1992).

[bib11] Baker Mona (1998).

[bib12] Battistella Edwin L. (2005).

[bib13] Bhais Eid (2011). https://core.ac.uk/reader/287328150.

[bib14] Díaz Cintas Jorge, Gambier Y., Gottlieb H. (2001). Multi) Media Translation.

[bib16] De Linde Zoe, Kay Neil (1999).

[bib17] Díaz Cintas Jorge, Remael Aline (2014).

[bib18] Fawcett Peter (1997).

[bib19] Furgani Taher (2016).

[bib20] Gamal Muhammad (2007). Audiovisual translation in the Arab world: a changing scene. Transl. Watch Quarterly.

[bib40] Hawel Zeineb Sami (2019). Strategies of subtitling swear words in the wolf of wall street movie. Lark Journl.

[bib21] Hermans Theo (1999).

[bib22] Jing-Schmidt Zhuo, Huang Chu-Ren, Jing-Schmidt Zhuo, Meisterernst Barbara (2019). Routledge Handbook of Chinese Applied Linguistics.

[bib23] Khalaf Abed Shahooth, Rashid Sabariah (2016). Attenuating obscenity of swearwords in the amateur subtitling of English movies into Arabic. Arab World Engl. J..

[bib24] Kruger Jan-Louis, Cintas Jorge Díaz (2008). The Didactics of Audiovisual Translation.

[bib25] Łabendowicz Olga (2012).

[bib26] Matkivska Nataliia (2014). Audiovisual translation: conception, types, characters’ speech and translation strategies applied. Kalbų studijos.

[bib27] Munday Jeremy (2009).

[bib28] Neves Josélia, Orero P. (2004). Topics in Audiovisual Translation.

[bib29] Newmark Peter (1988).

[bib30] Pardo Betlem Soler (2013). Translating and dubbing verbal violence in reservoir dogs. Censorship in the linguistic transference of quentin tarantino’s (swear) words. J. Special. Transl..

[bib31] Scott-Tennent Christopher, Davies Maria, Torras Fernanda (2000). Investigating Translation.

[bib32] Skuggevik Erik, Anderman G. (2009). Audiovisual Translation Language Transfer on Screen.

[bib33] Thawabteh Mohammad (2012). The translatability of euphemism and dysphemism in Arabic-English subtitling. Lexis: Journal in English Lexicology.

[bib34] Thawabteh Mohammad. (2017). Censorship in English-Arabic subtitling. Int. J. Transl..

[bib35] Tomaszkiewicz Teresa, Bogucki, Kredens K. (2010). *Perspectives on Audiovisual Translation,* Frankfurt/M.

[bib36] Toury Gideon (1995).

[bib37] Versteegh Kees (1997).

