# Erratum to “Subtitling Arabic profanities into English and that aggro: the case of West Beirut”<[Heliyon 8 (12) (2022) e11953]>

**DOI:** 10.1016/j.heliyon.2022.e12444

**Published:** 2022-12-19

**Authors:** Mohammad Ahmad Thawabteh, Amer Al-Adwan, Amna Shqair

**Affiliations:** a Al-Quds University, Jerusalem, Palestine; b Hamad bin Khalifa University, Qatar

In the original published version of this article, typesetting errors led to the incorrect appearance of Arabic text in Texts 1 - 10. This has now been corrected. The publisher apologizes for these errors. Both the HTML and PDF versions of the article have been updated to correct the errors.

